# Exciting High-Order Plasmon Mode Using Metal-Insulator-Metal Bowtie Nanoantenna

**DOI:** 10.3390/nano15120882

**Published:** 2025-06-07

**Authors:** Xiaoxin Zhang, Rulin Guan, Qingxiu Ding, Chen Wang, Yaqiong Li, Dengchao Huang, Qigong Chen, Zheng Yang

**Affiliations:** 1 Key Laboratory of Advanced Perception and Intelligent Control of High-End Equipment, Ministry of Education, School of Integrated Circuits, College of Electrical Engineering, Anhui Polytechnic University, Wuhu 241000, China; zhangxiaox9090@163.com (X.Z.); guanrulin@ahpu.edu.cn (R.G.); 2220320104@stu.ahpu.edu.cn (Q.D.); 18755062170@163.com (Y.L.);; 2 School of Internet of Things Engineering, Jiangnan University, Wuxi 214122, China; 3 Anhui Engineering Research Center of Vehicle Display Integrated Systems, School of Integrated Circuits, Anhui Polytechnic University, Wuhu 241000, China; 4 School of Robot Engineering, Anhui San Lian University, Hefei 230000, China; zy85618683@163.com

**Keywords:** LSPR, notch metal-insulator- metal bowtie nanoantenna, high-order plasmon modes, SERS

## Abstract

Noble metal nanostructures have garnered significant attention for their exceptional optical properties, particularly Localized Surface Plasmon Resonance (LSPR), which enables pronounced near-field electromagnetic enhancements. Among these, bowtie nanoantennas (BNAs) are distinguished by their intense plasmonic coupling within nanogap regions, making them highly effective for applications such as surface-enhanced Raman scattering (SERS). However, the practical utility of conventional BNAs is often hindered by small hotspot areas and significant scattering losses at their peak near-field enhancement wavelengths. To overcome these limitations, we have designed a novel notch metal-insulator-metal bowtie nanoantenna (NMIM-BNA) structure. This innovative design integrates dielectric materials with Ag-BNA nanostructures and strategically positions arrays of silver (Ag) nanorods within the central nanogap. By coupling the larger NMIM-BNA framework with these smaller Ag nanorod arrays, higher-order plasmon modes (often referred to as dark modes) are effectively excited. Consequently, the NMIM-BNA exhibits substantial electric field enhancement, particularly at the Fano dip wavelength, arising from the efficient coupling of these higher-order plasmon modes with dipole plasmon modes. Compared to conventional Ag-BNA nanoantennas, our NMIM-BNA provides a significantly larger hotspot region and an enhanced near-field amplification factor, underscoring its strong potential for advanced SERS applications.

## 1. Introduction

Typically comprising two triangular metallic elements positioned apex-to-apex with a nanoscale gap at their tips, the Bowtie Nanoantenna (BNA) harnesses Localized Surface Plasmon Resonance (LSPR) and strong tip-to-tip coupling to achieve substantial local electromagnetic field enhancement [1]. This characteristic makes BNAs highly effective for applications such as surface-enhanced Raman scattering (SERS) [2,3], optical sensing [4], single-molecule detection [5,6], and catalysis [7,8]. What distinguishes BNAs from other plasmonic nanostructures is their structural versatility and ability to generate efficient electromagnetic hotspots. Parameters including triangle size, tip angles, gap width, and material composition can be tailored to tune resonance frequencies across the visible to near-infrared spectrum while maximizing field enhancement within the nanogap. Additionally, their high directionality and superior optical focusing capabilities improve sensitivity and signal-to-noise ratios in various sensing and imaging applications [9,10]. However, the enhancement primarily depends on strong dipole resonance across the nanogaps, a phenomenon directly influenced by the gap size [11]. While smaller gap sizes amplify dipole–dipole interactions (bright modes), they also incur significant scattering losses at near-field enhancement peaks. Furthermore, gap sizes below a critical threshold may trigger electron tunneling between the metal nanostructures [12], demonstrating that reducing structural volume does not indefinitely improve electric field enhancement. The confined and non-uniform SERS hotspots in metallic nanostructures further limit the practical utility of metallic nanogap SERS substrates and other plasmonic devices, necessitating further optimization of nanostructure design to enhance the performance and applicability of plasmonic devices.

Recent studies on Localized Surface Plasmon Resonances (LSPRs) emphasize their role as nano-optical resonators [13]. A critical performance metric for such resonators is the Q/V ratio, which reflects the intensity of light confinement and correlates directly with near-field enhancement. Here, Q represents the quality factor (determined by the resonance’s energy confinement), and V denotes the mode volume, indicating the degree of near-field localization. While BNAs exhibit strong near-field enhancements due to dipole–dipole coupling in ultrasmall nanogaps, this coupling is typically associated with low Q-factors and substantial scattering losses [14]. For stronger near-field enhancement, optimization of both the mode volume (V) and quality factor (Q) is crucial. Unlike bright modes (e.g., dipole–dipole coupling), dark plasmon modes (e.g., quadrupole modes) can exhibit higher Q-factors [15], enabling superior near-field enhancement through high-Q plasmonic modes within relatively small nanogaps. Metal-insulator-metal (MIM) nanostructures are particularly effective at inducing dark plasmon modes by breaking structural symmetry [16]. The integration of dielectric materials and deliberate symmetry disruption in metallic nanostructures allows for the excitation of high-Q plasmonic dark modes [17]. Numerical simulations have revealed that MIM plasmonic bowtie resonators with a structured top metal layer can support two efficiently excitable gap surface plasmon (GSP) resonances, dominated by dipole and quadrupole moments, leading to distinct low- and high-Q factor responses, respectively, with the latter achieving Q~25 in the near-infrared [18].

In this study, we developed notch metal-insulator-metal bowtie nanoantennas (NMIM-BNAs) to achieve advanced electric field enhancement. By horizontally integrating insulating materials into conventional Ag-BNA nanostructures and strategically positioning multiple notched silver nanorods within the nanogap region, we disrupted the geometric symmetry of the nanophotonic structure. This design successfully excited hybrid dark plasmon modes with high Q-factors, enabling efficient coupling between the electric dipole resonance (a bright mode) and higher-order electric multipole moments (dark modes). The result was a significant electric field enhancement factor, particularly at the Fano dip wavelength. We systematically analyzed factors affecting this near-field enhancement, including structural dimensions, the proportion of insulating materials, material properties, and the number of nanorod arrays. Compared to conventional Ag bowtie nanoantennas, the hybrid NMIM-BNA structure demonstrated superior near-field performance, an expanded hotspot area, and reduced scattering losses. These improvements establish NMIM-BNAs as a highly promising candidate for SERS substrates and other advanced plasmonic applications.

## 2. Results and Discussion

### 2.1. Method

Our study investigates the electromagnetic properties of the proposed notch metal-insulator-metal bowtie nanoantenna (NMIM-BNA), focusing on its spectral behavior in both near- and far-field regions, as well as the spatial and temporal distribution of the near-field intensity. To analyze the optical behavior, the NMIM-BNA was excited by a plane wave incident perpendicularly to its short axis. The simulations were conducted using the three-dimensional finite-difference time-domain (FDTD) method implemented in CST Studio 2023. A minimum grid size of 0.4 nm was employed to ensure high-resolution calculations, and “open (add space)” boundary conditions were applied to support accurate far-field computations. The model incorporated realistic material parameters for silver (Ag), the dielectric material germanium (Ge), and air. An excitation wavelength range of 400–1000 nm was chosen to comprehensively study the electromagnetic responses over a broad spectral range. This approach enabled a detailed analysis of the NMIM-BNA’s electromagnetic behavior, capturing variations in angular modes and near-field intensity distributions. Specifically, the simulations recorded the total-field/scattered-field (TFSF) response of the inhomogeneous NMIM-BNA, providing critical insights into field confinement and enhancement by tracking key electromagnetic field components.

**Figure 1:**
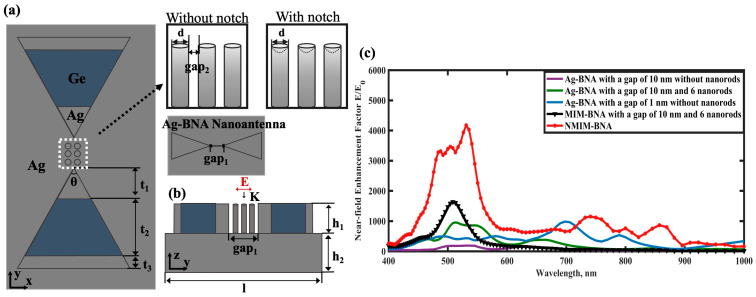
Structure of the MIM-BNA and CST simulation results for the near-field. (**a**) Top view and (**b**) vertical view of the MIM-BNA and NMIM-BNA; (**c**) Maximum electric field enhancement factor for the MIM-BNA, NMIM-BNA, and several similar BNA nanoantenna reference objects.

In the NMIM-BNA design, insulating materials (Ge) were horizontally integrated into conventional Ag-BNA nanostructures, and multiple silver nanorods were strategically positioned within the nanogap. The underlying MIM-BNA structure (without notched nanorods) can successfully excite dark plasmon modes by coupling the electric dipole resonance (a bright mode) with higher-order electric multipole moments of the broadband gap (dark modes), characterized by a high quality factor (Q-factor). This establishes the MIM-BNA as a promising nanostructure for achieving enhanced near-field electric field effects. To further amplify these effects, the NMIM-BNA was introduced, featuring notched Ag nanorods within the nanogap of the MIM-BNA. By disrupting the geometric symmetry, the NMIM-BNA design effectively enhanced the excitation of these dark plasmon modes. Comparative analyses showed that the NMIM-BNA significantly outperformed the MIM-BNA in near-field enhancement, as illustrated in Figure 1b. The structural details of the BNA and its variations are depicted in Figure 1a. The BNA consists of two symmetrical isosceles triangles with an apex angle *θ* and a total length *t* (nm). In the MIM-BNA configuration, *t* is the sum of *t*_1_, *t*_2_, and *t*_3_. The triangular MIM-BNA structure comprises Ag, Ge, and Ag layers with respective lengths *t*_1_, *t*_2_, and *t*_3_, and a height *h*_1_, which is equal to the base height *h*_2_. The nanorods embedded in the BNA’s nanogap are characterized by a number *N*, with each rod having a diameter *d* and a height matching that of the BNA. The nanogap width was set to 10 nm, with a spacing of 1 nm between the nanorods (gap_2_ = 1 nm). The optical properties of Ag and Ge were adopted from Palik’s dataset [19]. The fabrication process for MIM-BNA nanoantennas is detailed in references [20,21], involving focused ion beam (FIB) nanofabrication and electron beam lithography (EBL) to create large-scale hybrid dimer structures. Template-assisted methods were employed to prepare the nanorods integrated within these nanoantennas. Additionally, reference [22] describes a wafer-scale fabrication technique that achieves uniform sub-5-nanometer gaps through a combination of rapid dry etching and advanced lithography methods. The primary fabrication challenge lies in precisely controlling the notches on the nanorods and the gap width between them. Key structural parameters are summarized in Table 1.

In order to further improve the application prospects of this type of SERS substrate, we also discussed the manufacturing process of this type of SERS substrate. The fabrication problem of this structured SERS mainly focuses on how to place the silver nanorod array in the MIM-BNA nanostructures using appropriate technology. etch lateral Ag nanowires on the substrate. FIB-assisted deposition is used to inject Ge precursors (such as Ge(CH_3_)_4_) into the Ag gap to form a lateral dielectric layer. Ag is deposited on the other side of the Ge layer by FIB to complete the lateral Ag-Ge-Ag bowtie nanostructure. Then, metallic nanorods (diameter: 2–250 nm) are synthesized via electroless plating or electrochemical deposition using porous anodic aluminum oxide or polycarbonate Use SEM mode to locate the target nanorod and set the Ga⁺ beam current to scan a specific pattern (e.g., circle or square) on the top of the nanorod to sputter and remove material to form holes. We can control the size and depth of the aperture by adjusting the beam dose and scan time. Subsequently, gradient centrifugation isolates notch nanorods of target dimensions. Finally, Photolithography is employed to define gap regions on the substrate, after which dispersed nanorod solutions are drop-casted into these predefined gaps. Electric field-assisted assembly or directed self-assembly techniques align the nanorod arrays within the gaps, ultimately yielding MIM-BNA nanostructures integrated with precisely positioned nanorod arrays. This hierarchical approach enables sub-5 nm gap precision and enhanced plasmonic coupling, as validated by Transmission Electron Microscopy and finite-difference time-domain simulations in recent studies.

### 2.2. Numerical Simulation of the Near-Electric Field

**Figure 2:**
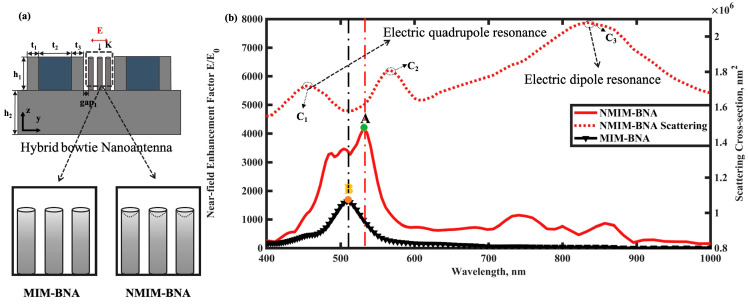
Structure of the MIM-BNA and CST simulation results for the near-field and far-field. (**a**) vertical view of the MIM-BNA and NMIM-BNA; (**b**) Maximum electric field enhancement factor for the MIM-BNA, NMIM-BNA, and scattering cross-section of Hybrid bowtie nanoantennas. Supplementary discussions of plasmon resonance mechanisms have been incorporated at designated wavelengths (A, B, C1, C2).

CST simulations confirmed the near-field enhancement effects of the Metal-Insulator-Metal Bowtie Nanoantenna (MIM-BNA) and the Notched MIM-BNA (NMIM-BNA). Figure 2b illustrates the near-field enhancement and scattering intensity profiles of both structures across various wavelengths, with their respective peak near-field electric enhancements labeled A (for NMIM-BNA) and B (for MIM-BNA). In conventional all-metal BNA designs, the maxima of scattering intensity and electric field enhancement typically coincide due to the excitation of electric dipole resonance. In contrast, for both our hybrid NMIM-BNA and MIM-BNA nanostructures, the maximum electric field enhancement is notably observed near a minimum in the scattering intensity. This characteristic phenomenon is attributed to the efficient excitation of dark plasmon modes within these hybrid architectures. Our findings reveal that the MIM-BNA structure alone achieves a near-field enhancement factor several times greater than that of conventional all-metal BNA structures. Furthermore, the near-field enhancement capability of the MIM-BNA is substantially improved by introducing notched nanorods, as realized in the NMIM-BNA design. Compared to the MIM-BNA, the NMIM-BNA structure demonstrates superior near-field enhancement performance across a broader wavelength range. These results underscore the effectiveness of material hybridization and nanostructure notching in significantly amplifying near-field electric field intensity while simultaneously minimizing light scattering losses.

To further analyze the plasmon resonance modes in this structure, we compare the near-field enhancement effects of two nanostructures: Ag-Notch Bowtie nanoantenna and NMIM-BNA, as shown in Figure S1 illustrates the near-field enhancement effects and scattering intensities of both the NMIM-BNA and NAg-NBNA across various wavelengths. Typically, in Ag-NBNA configurations, the maxima of both the scattering field and the electric field enhancement are observed at identical wavelengths, characteristic of electric dipole resonance excitation. In this study, while the near-field enhancement peaks for both NAg-NBNA and NMIM-BNA occur around 530 nm, their corresponding scattering cross-sections at this wavelength differ significantly: the NMIM-BNA’s scattering is approximately 85% of the NAg-NBNA’s. Furthermore, a key distinction is that the NAg-NBNA’s enhancement peak coincides with its scattering cross-section peak, whereas the NMIM-BNA’s enhancement peak aligns with a scattering trough. Notably, the scattering intensity of the NMIM-BNA at its near-field enhancement location (530 nm) is reduced by 30% compared to its main scattering peak (observed around 800 nm). This observation further supports the concept that incorporating high-refractive-index materials can alter their plasmon resonance modes, leading to Fano-like resonance phenomena and a broadened wavelength range for near-field enhancement.

To elucidate the plasmon resonance modes of the structure at specific wavelengths (C1, C2, C3, and the near-field enhancement peak A), we employed a multipole decomposition method based on the scattered energy ratio. This approach first involves special processing of the electric field vector data from the near-electric field distributions calculated by 2023 CST simulation software at the specific wavelengths, creating a dataset of electric field vectors with corresponding coordinate information. Subsequently, this electric field vector dataset is imported to calculate relevant field parameters, and the contribution rates of various multipoles (such as electric dipole (ED), magnetic dipole (MD), toroidal dipole (TD), electric quadrupole (EQ), and electric octupole (EO)) are determined according to established formulas, as summarized in Table 2. At wavelength C3, the scattering response is clearly dominated by the electric dipole (ED), with a contribution exceeding 91%, indicative of a strong bright mode resonance. At wavelength C1, the electric quadrupole (EQ) emerges as the dominant contributor to scattering (54%), with a relatively smaller ED contribution (20%), signifying a mode with strong quadrupolar characteristics. Wavelength C2 exhibits a more complex, mixed-mode scattering profile. Here, the electric dipole (ED) contribution (39%) is slightly larger than the electric quadrupole (EQ) contribution (35%), while significant contributions from the toroidal dipole (TD, 10%) and electric octupole (EO, 14%) are also observed. This indicates a hybrid mode where multiple multipolar orders contribute to the far-field scattering. Notably, at wavelength A, which corresponds to the maximum near-field enhancement, the mode is characterized by a dominance of non-dipolar contributions. The electric quadrupole (EQ, 37%) is the largest single contributor, followed by the electric octupole (EO, 23%) and a notable toroidal dipole (TD, 11%) contribution. The electric dipole (ED) contribution is relatively suppressed at 22%. The collective contribution of these higher-order (EQ, EO) and inherently low-radiating (TD) multipoles (totaling 78% of scattered energy) imparts significant subradiant or “dark-mode-like” characteristics to this resonance. This reduced far-field radiation efficiency facilitates strong energy confinement in the near-field, which is crucial for the observed strong local field enhancement at point A. Furthermore, our calculations indicate that the contributions from the magnetic dipole (MD) are relatively low across most of these wavelengths, except for a minor contribution at point A (7%). Overall, the distinct multipolar signatures at C1, C2, C3, and A highlight the rich modal landscape of the NMIM-BNA structure.

**Figure 3:**
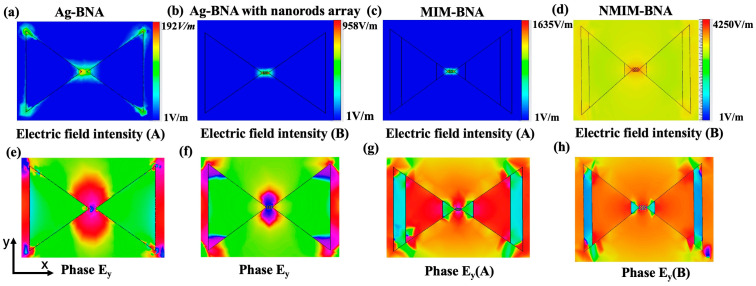
Near-electric field result: (**a**–**d**) the near-electric field peak distributions of Ag-BNA, Ag-BNA with nanorods array, MIM-BNA, and NMIM-BNA. (**e**–**h**) the electric field phase information of Ey (at y-z plane) at the wavelengths of near-field peaks.

This study indicates that the electric field amplification of the NMIM-BNA undergoes a significant increase within a particular wavelength spectrum, with the maximum electric field enhancement precisely positioned adjacent to a minimum in the scattered intensity. Concurrently, the insertion of a silver nanorod array within the gap successfully couples the dipole resonance of the nanorod array with the higher-order plasmon resonance mode of the MIM-BNA structure, achieving a significant electric field enhancement effect at the scattering trough. This phenomenon is closely related to the dark plasmon modes excited in such hybrid notched nanostructures. This demonstrates the effectiveness of utilizing gap engineering to significantly enhance near-field electric field intensity while also, to some extent, reducing the scattering intensity at the near-field enhancement peak. Further analysis reveals that this resonance effect not only improves the local electric field strength in the near field but also optimizes the scattering characteristics of light to a certain degree, providing a theoretical basis for enhancing sensor sensitivity. Figure 3a–d presents the maximum near-field electric distributions (y–z plane) for the nanostructures at their peak electric field wavelengths. These distributions provide a clear comparison of the effectiveness of the nanostructures and highlight differences in their electric field enhancement properties. In addition, apart from the Ag-BNA, the electric field phases within the hotspot regions exhibit a phase difference of approximately 180 degrees, as depicted in Figure 3e–h. Ag-BNA structures excite in-phase modes driven by strong dipole–dipole interactions, whereas MIM-BNA structures induce out-of-phase modes (dark plasmon modes) with higher Q factors, resulting in enhanced electric field strength. The hotspots in Ag-BNA structures are predominantly concentrated at the tip regions. In contrast, the NMIM-BNA exhibits more concentrated and distinct hotspots within the nanogap, indicating its superior optical response at the peak wavelengths. Furthermore, the integration of dielectric materials into the nanorods significantly modifies the optical response of the NMIM-BNA structure, extending its near-field enhancement effects into the near-infrared region.

**Figure 4:**
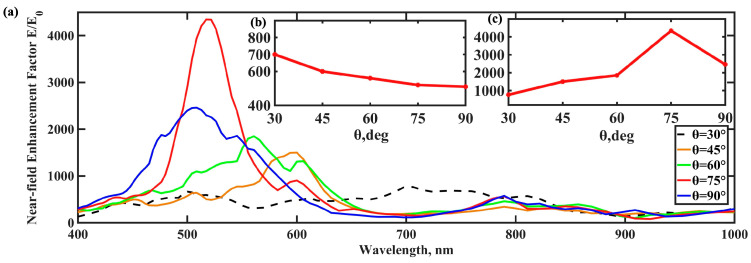
(**a**) the tip angle (*θ*) dependence of the near-field enhancement factor for NMIM-BNA structures. (**b**) Effect of tip angle (*θ*) in NMIM-BNA structures on maximum near-field enhancement. (**c**) Relationship between the peak wavelength of electric field enhancement and the tip angle (*θ*) in NMIM-BNA structures.

The near-field enhancement in BNA nanostructures is generally attributed to the “tip enhancement effect” generated by the metal nanogap. To investigate its influence on near-field enhancement in our system, we varied the tip angle (θ) of the metallic components in the MIM-BNA structures, as illustrated in Figure 4a. The results, presented in Figure 4c, reveal a strong correlation between the tip angle and near-field enhancement: the enhancement factor increases steadily as θ grows, reaching its maximum at 75 degrees. Specifically, compared to a tip angle of 30 degrees, the near-field enhancement factor of the NMIM-BNA structure at 75 degrees increased by approximately eightfold, demonstrating significant optimization potential. This nonlinear behavior is likely attributed to the increased proportion of insulating material within the gap region as θ enlarges, which, in turn, influences the electric field distribution and local field intensity. As the metal tip angle approaches a critical value, anomalous focusing and enhanced electromagnetic coupling effects can become more pronounced, resulting in substantial changes to the near-field enhancement. To further explore this relationship, we analyzed the connection between the peak enhancement wavelength and θ, as depicted in Figure 4b. As θ increases, the peak enhancement wavelength undergoes a blueshift, transitioning from the near-infrared region towards the visible spectrum. This behavior contrasts with that of conventional all-metallic BNA structures, where localized electric field enhancement is predominantly driven by dipole resonance, leading to enhancements mainly concentrated within the near-infrared range.

We conducted a comprehensive analysis of how the occupancy and position of the dielectric layer in NMIM-BNA structures influence electric field enhancement using CST simulation software. Our simulations initially focused on evaluating the effect of varying dielectric layer occupancies on electric field enhancement in this nanostructure. Figure 4a illustrates the electric field enhancement effects for NMIM-BNA at dielectric layer occupancies of 0%, 30%, 50%, 70%, and 100%, respectively. When maintaining the metal-dielectric-metal structure, even with no explicit dielectric layer, the near-field enhancement factor of the NMIM-BNA gradually increases as the proportion of the intentionally introduced dielectric material rises. Notably, when the dielectric layer occupancy reaches 50%, the near-field enhancement factor increases significantly. This phenomenon may result from the dielectric layer’s thickness equaling the combined thickness of the metal layers on either side, potentially causing the electric field phase E_y_ to reverse on those opposing sides. Additionally, as the proportion of the dielectric layer increases, the peak wavelength of the near-field enhancement factor undergoes a gradual blueshift. The structure also exhibits at least two near-field enhancement peaks, located in the blue and near-infrared spectral regions, respectively.

**Figure 5:**
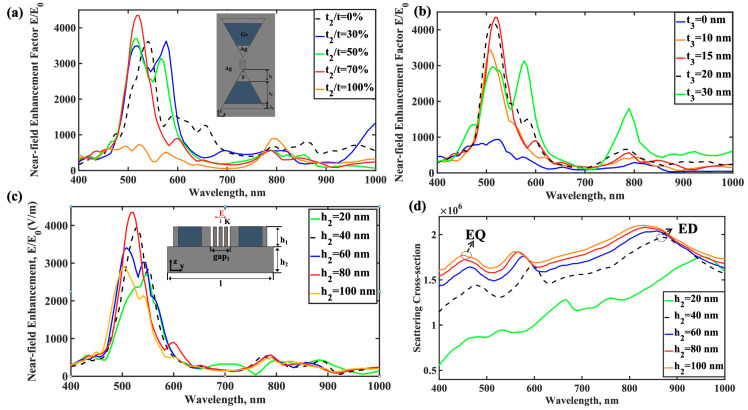
CST simulation results. (**a**) Electric field enhancement characteristics of NMIM-BNA with dielectric layer occupancy (*t*_2_*/t*) from 0% to 100%. (**b**) Effect of the position of the dielectric layer (*t*_3_) on the electric field enhancement of NMIM-BNA. Effect of NMIM-BNA nanostructure basal layer thickness *h*_2_ on electric field enhancement (**c**) and scattering cross-section (**d**).

We also analyzed the impact of positional changes of the dielectric layer on the electric field distribution. Figure 4b shows the electric field enhancement effect for the NMIM-BNA with different dielectric layer positions. We set t_1_ + t_2_ + t_3_ = 100 nm and h_1_ = h_2_ = 80 nm. When t_3_ = 0 nm, the nanostructure forms a metal-dielectric bilayer hybrid structure. In this configuration, the near-field enhancement factor is relatively low, influenced by the dipole resonance mode of the nanorod array in the nanogap (around 800 nm wavelength). However, as the dielectric layer shifts toward the outer edge of the BNA structure, the increased metal layer thickness at the NMIM-BNA tip results in a near-field enhancement factor more than four times greater than that of the initial metal-dielectric BNA structure (t_3_ = 0 nm). Additionally, when t_3_ = 30 nm, the dielectric-metal BNA structure exhibits three distinct near-field enhancement peaks at wavelengths of approximately 500 nm, 590 nm, and 800 nm. The peaks at the two longer wavelengths are attributed to the excitation of electric quadrupole and dipole resonances, respectively, while the shorter-wavelength peak results from the excitation of a new dark plasmon mode. As shown in Figure 5c, the near-field enhancement factor also increases with both the substrate thickness and the refractive index of the dielectric layer. An increase in substrate thickness causes a shift in the peak wavelength of the near-field enhancement. In the NMIM-BNA structure, as the substrate thickness increases, the electromagnetic field becomes more localized within the nanogaps. This enhanced localization is proposed to facilitate energy transfer between the electric dipole resonance mode and the electric quadrupole resonance mode, resulting in a blueshift of the scattering peak, as depicted in Figure 5d. Furthermore, our multipole decomposition analysis clearly reflects the evolution of plasmon resonance modes at the scattering peaks, revealing that both the electric dipole (ED) and electric quadrupole (EQ) contributions exhibit a progressive blueshift with increasing substrate thickness.

**Figure 6:**
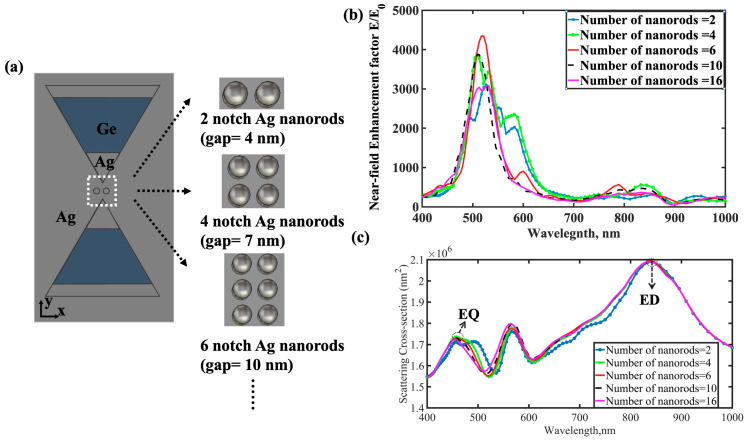
(**a**) CST simulation model of MIM-BNA on an Ag substrate; (**b**) electric field enhancement and (**c**) scattering cross-section of NMIM-BNA structures as the number of nanogap nanorods varies from 2 to 16.

The spatial extent of the “hotspot” region and the electric field enhancement factor in NMIM-BNA structures are strongly influenced by the number of Ag nanorods present within the nanogap. Using CST simulation software, we analyzed how variations in the number of Ag nanorods (N) affect these properties. For conventional Ag-BNA structures, the maximum electric field enhancement decreases significantly as the nanogap width increases, reflecting the strong dependence of dipole–dipole interactions on the gap width. In our NMIM-BNA nanoantennas, when the number of Ag nanorods ranges from 2 to 8, the near-field enhancement factor increases significantly, as shown in Figure 6b. Interestingly, the peak electric field enhancement in the NMIM-BNA does not occur at the narrowest inter-rod gap but rather when the overall gap occupied by the nanorod array is approximately 10 nm. As the number of nanorods increases, the scattering peak of the structure undergoes a noticeable blueshift. Moreover, the peak of the maximum near-field enhancement factor consistently coincides with a trough in the scattering field, as depicted in Figure 6c. This finding suggests that the electric field enhancement in NMIM-BNA structures does not originate solely from dipole resonance but instead results from the excitation of higher-order dark plasmon modes.

To contextualize the performance of our proposed NMIM-BNA structure, a comparison with representative state-of-the-art (SOTA) SERS substrates is warranted. Numerically, the NMIM-BNA architecture predicts significant near-field enhancement capabilities, with calculated SERS enhancement factors in the range of 10^13^–10^14^. This level of enhancement is highly competitive with, or comparable to, many contemporary SERS platforms, including those based on nanoparticle-on-film configurations (typically EFs~10^10^–10^12^) [23] and some lithographically defined narrow-gap arrays [24]. While certain specialized SOTA systems, particularly those leveraging angstrom-scale gaps for single-molecule SERS, may report even higher localized EFs, our NMIM-BNA design focuses on achieving a robust average enhancement over an extended and engineered active area. Regarding hotspot characteristics, the NMIM-BNA offers a distinct advantage over substrates relying on random nanoparticle aggregation by providing an engineered distribution and potentially higher effective density of hotspots, facilitated by the defined nanorod array within the bowtie gap. When compared to some advanced, large-area planar SERS substrates produced by precision nanopatterning, the latter might offer a greater overall active surface. However, the NMIM-BNA prioritizes extreme field localization and enhancement within multiple, well-defined sub-volumes created by the nanorod array. This targeted enhancement in confined regions can be particularly beneficial for sensing applications requiring high sensitivity within specific interrogation volumes.

Regarding hotspot characteristics, the NMIM-BNA offers a distinct advantage over substrates relying on random nanoparticle aggregation by providing an engineered distribution and potentially higher effective density of hotspots, facilitated by the defined nanorod array within the bowtie gap. When compared to some advanced, large-area planar SERS substrates produced by precision nanopatterning, the latter might offer a greater overall active surface. However, the NMIM-BNA prioritizes extreme field localization and enhancement within multiple, well-defined sub-volumes created by the nanorod array. This targeted enhancement in confined regions can be particularly beneficial for sensing applications requiring high sensitivity within specific interrogation volumes. In terms of reproducibility, the deterministic, lithography-oriented design of the NMIM-BNA inherently offers a pathway to superior consistency compared to stochastically formed SERS substrates, which often exhibit significant signal variability. Achieving high reproducibility is a critical challenge in the SERS field, with many SOTA approaches also focusing on top-down fabrication for better control. While our proposed fabrication pathway aims for a deterministic hotspot arrangement, we acknowledge that, like many plasmonic structures utilizing silver, long-term performance reproducibility will necessitate further investigation into strategies for mitigating metal surface oxidation, such as surface passivation or the integration of more stable plasmonic materials. Addressing this aspect remains an important direction for future optimization to fully realize the reproducibility potential of such rationally designed SERS substrates.

## 3. Conclusions

Nanogap nanoantennas, such as bowtie antennas (BNAs), are renowned for their exceptional near-field enhancement driven by Localized Surface Plasmon Resonances (LSPRs). These antennas typically achieve high near-field enhancement through strong dipole–dipole interactions (plasmon bright modes) within a small nanogap; however, this is often accompanied by significant radiation losses and limited hotspot areas. To address these limitations, we propose a novel design strategy to simultaneously enhance near-field effects, mitigate radiation losses, and expand hotspot areas. This approach involves embedding multiple notched silver nanorods within the nanogap of Metal-Insulator-Metal Bowtie Nanoantenna (MIM-BNA) nanostructures. This approach successfully coupled bright (dipole mode of the nanorod array) and dark plasmonic modes (EQ mode of the MIM-BNA), enhancing the electric field at the Fano dip wavelength. Using CST simulation software, we systematically analyzed the impact of key design parameters on the near-field electric field enhancement in these NMIM-BNA structures. Our design achieves a near-field enhancement factor approximately 20 times greater than that of conventional Ag-BNA structures, over a broader optical frequency range and with larger hotspot areas. This substantial improvement is primarily attributed to the efficient excitation of higher-order plasmonic modes possessing significantly higher Q-factors. Our findings provide a highly flexible and efficient nanostructured solution for Surface-Enhanced Raman Scattering (SERS) applications across various wavelength ranges.

## Figures and Tables

**Table 1:** Specific parameters of NMIM-BNA, MIM-BNA, and different size parameters of Ag-BNA nanostructures.

Parameters	*Gap*_1_ (nm)	*θ* (deg)	*t* (nm)	*t*_1_ (nm)	*t*_2_ (nm)	*t*_3_ (nm)	*h*_1_ (nm)	*d* (nm)	*N*
NMIM-BNA	10	75	100	20	60	20	80	3	6
MIM-BNA	10	75	100	20	60	20	80	2	6
1 nm-Ag-BNA	1	75	100	/	/	/	80	/	/
10 nm-Ag-BNA	10	75	100	/	/	/	80	/	/

**Table 2:** Multipole decomposition of NMIM-BNA nanoantenna at various wavelength positions (C1, C2, C3, A).

Scattered Energy Ratio	C1	A	C2	C3
Electric Dipole (ED)	20%	22%	39%	91%
Electric Quadrupole (EQ)	54%	37%	35%	5%
Toroidal Dipole (TD)	2%	11%	10%	1%
Magnetic Dipole (MD)	2%	7%	2%	1%
Electric Octupole (EO)	13%	23%	14%	2%

## Data Availability

The original contributions presented in this study are included in the article/Supplementary Material. Further inquiries can be directed to the corresponding author.

## Supplementary Materials

The following supporting information can be downloaded at: https://www.mdpi.com/article/10.3390/nano15120882/s1, Figure S1: The near-field enhancement effects and scattering intensities of both the NMIM-BNA and NAg-NBNA across various wavelengths.
