# Supplementary figures and images for: Exciting High-Order Plasmon Mode Using Metal-Insulator-Metal Bowtie Nanoantenna
Source: Nanomaterials (Basel). 2025 Jun 7;15(12):882. doi: 10.3390/nano15120882 (PMC12195817; doi:10.3390/nano15120882)

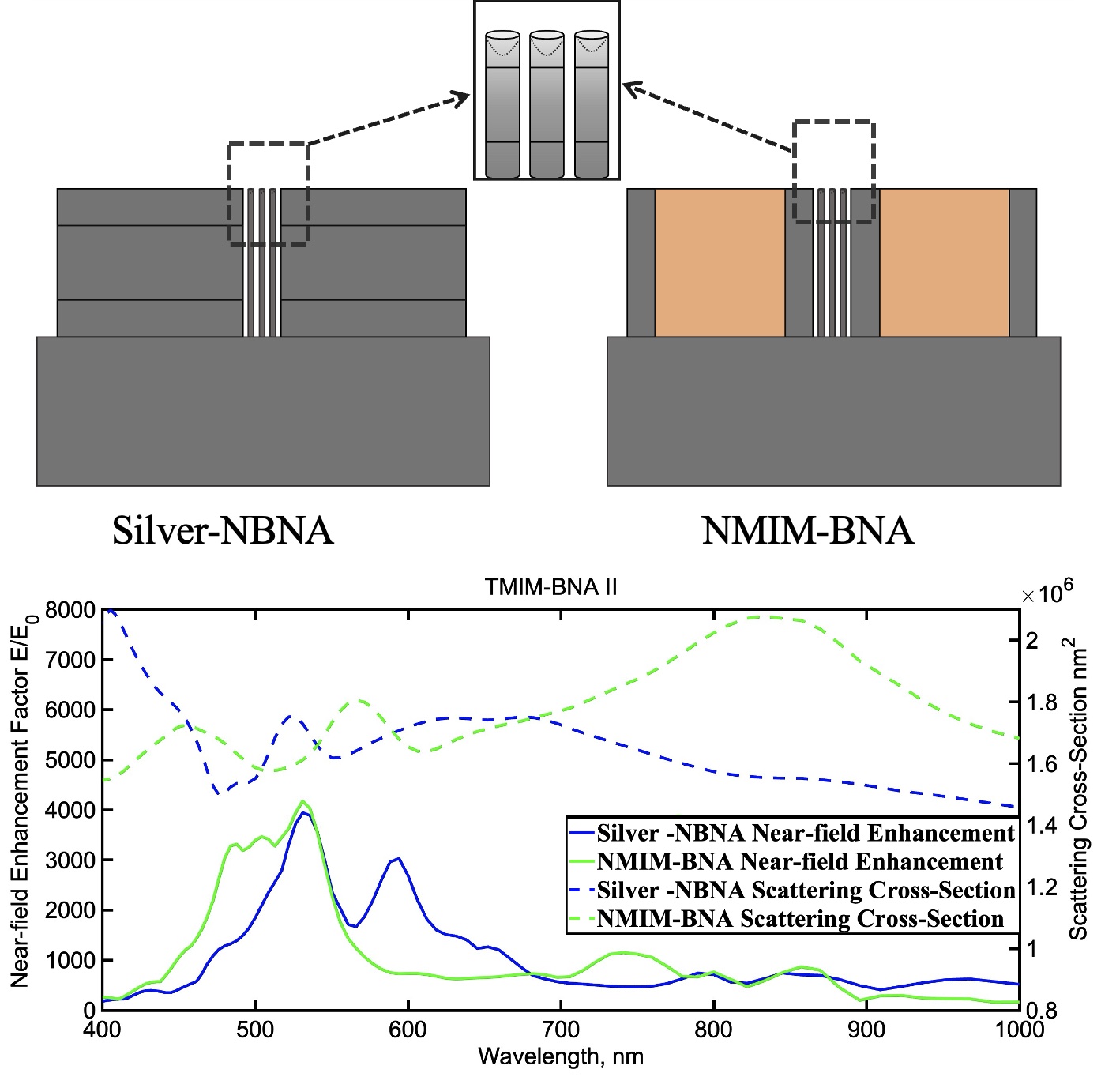

Supplement: Supplementary file 1 [file nanomaterials-15-00882-s001.zip › Figure S1.jpg]
